# Epidemiology of hip fracture in Belarus: development of a country-specific FRAX model and its comparison to neighboring country models

**DOI:** 10.1007/s11657-018-0454-4

**Published:** 2018-04-17

**Authors:** H. Ramanau, I. Chernyanin, E. Rudenka, O. Lesnyak, A. Zakroyeva, J. P. Bilezikian, H. Johansson, N. C. Harvey, E. V. McCloskey, J. A. Kanis

**Affiliations:** 1 Gomel State Medical University, 5 Lange Street, 246050 Gomel, Belarus; 2 Mozyr Central City polyclinic, 14a Kotlovtsa Street, 247760 Mozyr, Belarus; 3 Belarusian State Medical University, 83 Dzerzhinski Ave., 220116 Minsk, Belarus; 4 North West State Medical University named after I.I.Mechnikov, 41, Kirochnaya Street, St. Petersburg, 191015 Russia; 5 Ural State Medical University, 3 Repina Street, Yekaterinburg, 620028 Russia; 6 College of Physicians & Surgeons, Columbia University, 630 W. 168th Street, New York, NY 10032 USA; 7 Institute for Health and Ageing, Catholic University of Australia, Melbourne, Australia; 8 MRC Lifecourse Epidemiology Unit, University of Southampton, Southampton, UK; 9 Centre for Metabolic Bone Diseases, University of Sheffield, Sheffield, UK

**Keywords:** FRAX, Fracture probability, Epidemiology, Hip fracture

## Abstract

***Summary*:**

Fracture probabilities resulting from the newly generated FRAX model for Belarus based on regional estimates of the hip fracture incidence were compared with FRAX models of neighboring countries. Differences between the country-specific FRAX patterns and the rank orders of fracture probabilities were modest.

**Objective:**

This paper describes the epidemiology of hip fractures in Belarus that was used to develop the country-specific fracture prediction FRAX® tool and illustrates its features compared to models for the neighboring countries of Poland, Russia, and Lithuania.

**Methods:**

We carried out a population-based study in a region of Belarus (the city of Mozyr) representing approximately 1.2% of the country’s population. We aimed to identify all hip fractures in 2011–2012 from hospital registers and primary care sources. Age- and sex-specific incidence and national mortality rates were incorporated into a FRAX model for Belarus. Fracture probabilities were compared with those derived from FRAX models in neighboring countries.

**Results:**

The estimated number of hip fractures nationwide in persons over the age of 50 years for 2015 was 8250 in 2015 and is predicted to increase to 12,918 in 2050. The annual incidence of fragility hip fractures in individuals aged 50 years or more was 24.6/10,000 for women and 14.6/10,000 for men, standardized to the world population. The comparison with FRAX models in neighboring countries showed that hip fracture probabilities in men and women in Belarus were similar to those in Poland, Russia, and Lithuania. The difference in incidence rates between the surveys including or excluding data from primary care suggested that 29.1% of patients sustaining a hip fracture were not hospitalized and, therefore, did not receive specialized medical care.

**Conclusion:**

A substantial proportion of hip fractures in Belarus does not come to hospital attention. The FRAX model should enhance accuracy of determining fracture probability among the Belarus population and help guide decisions about treatment.

## Introduction

The growing worldwide burden of osteoporosis requires predictive tools for an individual fracture risk assessment so that appropriate intervention can be effectively targeted. Of the risk assessment tools available, the most widely used is FRAX® that was released in 2008 by the WHO Collaborating Centre at Sheffield (https://www.sheffield.ac.uk/FRAX/). At present, FRAX models are available for 63 countries. The FRAX tool computes age-specific fracture probabilities in women and men from readily obtained clinical risk factors (CRFs) and BMD measurements at the femoral neck. The algorithm (FRAX) was based on a series of meta-analyses using the primary data from population-based cohorts that identified several CRFs for fracture [1, 2]. FRAX models compute the probabilities of major osteoporotic and hip fracture derived from the risk of fracture and the competing risk of death, both of which vary from country to country [3].

To develop country-specific FRAX models, data relevant to both fracture incidence and death should be available [4, 5]. The aim of this study was to create a FRAX model for Belarus, using recently acquired population-based data of hip fracture rates and to compare this with FRAX models of neighboring counties Poland, Russia, and Lithuania.

## Methods

Belarus is a landlocked upper middle-income country in Eastern Europe with the total area of 207,600 km^2^ and a current population of 9,505,000. It is bordered by Russia, Ukraine, Poland, Lithuania, and Latvia [6]. More than 130 ethnic groups live in Belarus of which 83.7% are Belarusian, 8.3% Russian, 3.1% Polish, and 1.7% Ukrainian. WHO statistics indicate that the average life expectancy from birth in Belarus is 72 years, and individuals aged over 60 years account for 20% of the total population. Among the leading causes of death are non-communicable diseases, which account for 89% of all deaths [7].

The city of Mozyr (Gomel region) with a population of 129,724 in 2011 was selected to document the hip fracture rate. Mozyr is located far from the big Belarussian cities, which restricts the patients’ ability to seek medical care elsewhere and provided, therefore, a well-defined catchment population. According to the National Statistics Service, this region represents about 1.2% of the entire population of Belarus. Belarusians predominate as an ethnic group in Mozyr (89.5%), followed by Russians (6.6%). Thus, the ethnic distribution of the catchment population was comparable to that of Belarus. In 2011, the population aged 50 years and older amounted to 14,380 men and 21,069 women [8, 9]. The city has three outpatient polyclinics, a central city hospital, and an outpatient trauma unit that is open 24 h a day.

During the period of 2011–2012 (2 years), we undertook a prospective search for cases of hip fractures from all available sources, as well as their verification. The medical records of hip fractures in men and women aged 50 years or older were provided from the central city hospital registers, the outpatient trauma unit data, and emergency service data according to ICD-10 codes (S72.0, S72.1, S72.2). All the fracture cases were validated from radiographs. An additional source of information was obtained from official outpatient notes and records of the visits of orthopedic surgeons to patients at home.

In addition, we surveyed all community sources and general practices to capture all reliable data on hip fracture patients who might not have sought orthopedic care and, subsequently, were not registered in the orthopedic service records. We interviewed all 44 general practitioners from the three city outpatient hospitals of Mozyr to determine if there were poorly mobile or bedridden elderly persons who had sustained a hip fracture. According to the study protocol, an orthopedic surgeon subsequently examined such people at home. Where cases of hip fracture were suspected, the diagnosis was verified clinically. Where possible, these patients also underwent X-ray examination. The patient’s age, place of residence, sex, date, and character of injury were documented on case report forms.

All medical records were reviewed to check the place of residence, level of injury, and patients’ age. For each hip fracture site, we excluded a second admission in the observation time for the same fracture site. Pathological fractures and high energy fractures were also excluded (falls from greater than from a standing height). If the patient sustained a second hip fracture during the survey, it was registered as a new fracture. Only residents of Mozyr, aged 50 years and older, were included.

Incidence was computed by age and sex in 5-year age intervals using the population demography from Mozyr, supplied from the 2011 census. To compute the number of hip fractures in Belarus, age- and sex-specific incidence rates were applied to the population of Belarus for 2015 [10]. For comparison of hip fracture rates in Belarus with neighboring countries (Lithuania, Poland, and Russia), we used age- and sex-specific rates adjusted to the world population (2010) [3].

The development and validation of FRAX have been extensively described elsewhere [1, 3]. The incidence of other major osteoporotic fractures was not determined, and for the purposes of the FRAX model, it was assumed that the age- and sex-specific ratios of forearm, clinical spine, and humeral fracture incidence to hip fracture incidence in Sweden [11] were comparable to those in Belarus. This assumption has also been used for many of the FRAX models with incomplete epidemiological information. Available information suggests that the age- and gender-stratified pattern of fracture is very similar across countries [12]. For the construction of FRAX, we used data on mortality from the WHO web site [7] and population projections from 2015 from the Belarus State Statistics Committee [8].

## Results

For the period of 2 years (from 01.01.2011 to 31.12.2012), we recorded 117 cases of hip fracture, of whom 83 (70.9%) underwent in-patient treatment. Non-hospitalized patients were significantly older (mean age 82.3 ± 9.8 years vs. 71.8 ± 11.3 years, *p* < 0.001), and more likely to be female.

**Fig. 1:**
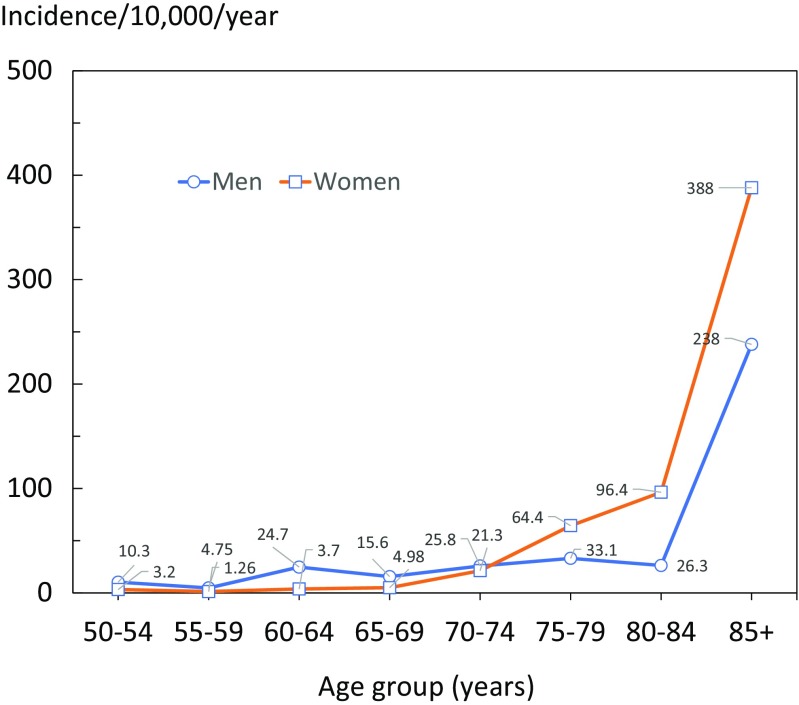
The annual incidence of hip fracture (rate/10,000) by age and sex in Belarus among women (squares) and men (circles)

The number of hip fractures in women was 76 and 41 in men (ratio 1.9/1). Hip fracture rates started to increase from the age of 60 years. The incidence in men younger than 70 years was higher than that of women, but among the most elderly residents of Mozyr, the hip fracture incidence in women was 60% higher than in men (Fig. 1). The total number of hip fractures in Belarus in 2015 was estimated at 8250 for a population of 9.45 million.

**Fig. 2:**
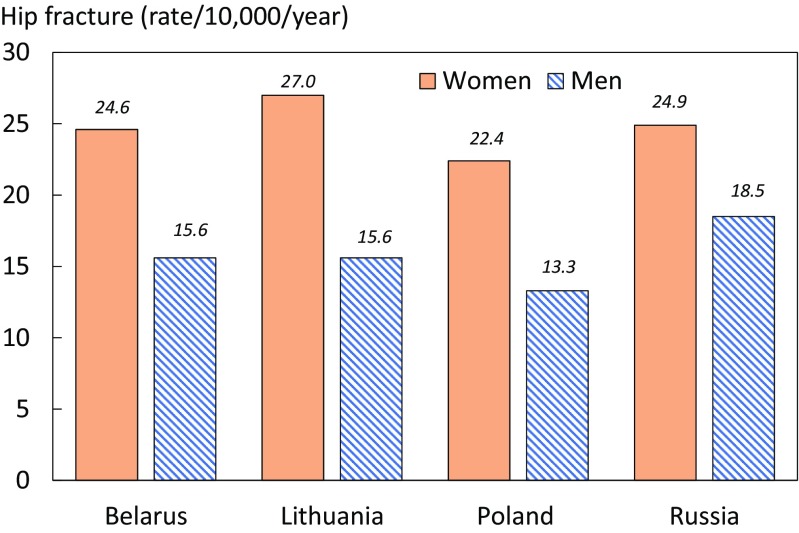
Comparison of world standardized hip fracture incidence in Belarus and neighboring countries

The world standardized annual hip fracture rate (/10,000) in men and in women is shown in Fig. 2 and compared with neighboring countries. The hip fracture incidence in women in Belarus was similar to that in Russia with slightly higher rates in Lithuania and lower rates in Poland.

### Fracture probability

The 10-year probability of a major osteoporotic fracture at the age of 50 years increased progressively with age in men and women. In men with no clinical risk factors (BMI set at 25 kg/m^2^ and the BMD not known), hip fracture probability rose from 0.1% at the age of 50 years to 4.6% at the age of 90 years; for women, the probabilities were 0.2 and 8.8%, respectively (Table 1). Fracture probabilities were approximately doubled in men and women with a prior fragility fracture.

**Table 1:** 10-year probability of a major osteoporotic fracture in men and women by age for individuals without clinical risk factors (CRFs) and in men and women with a prior fragility fracture. In both scenarios, BMI was set to 25 kg/m^2^, and without BMD

Age (years)	No CRFs		Prior fracture	
	Men	Women	Men	Women
50	1.6	2.2	3.4	4.9
55	1.5	2.4	3.2	5.2
60	1.4	2.5	2.9	5.2
65	1.5	3.1	3.1	6.2
70	2.0	4.6	4.0	8.9
75	3.1	7.9	5.7	14
80	4.7	12	7.7	19
85	6.1	15	9.8	24
90	7.5	18	12	27

**Fig. 3:**
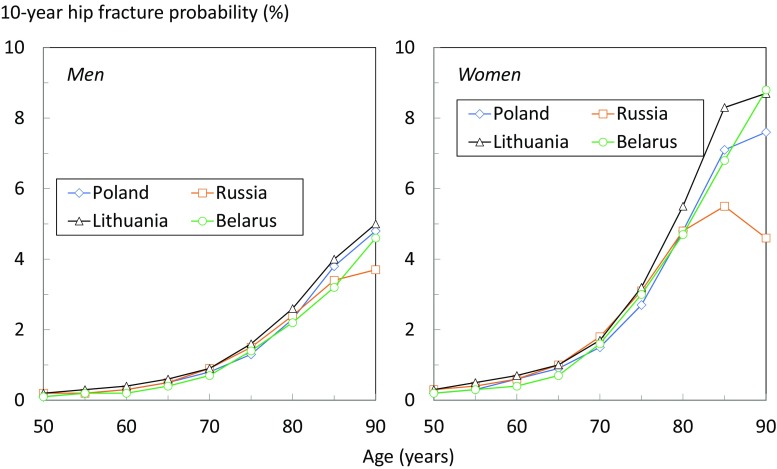
10-year probability of hip fracture in men (left panel) and women (right panel) by age for individuals without clinical risk factors, a BMI set to 25 kg/m^2^, and without BMD, in Belarus, Poland, Russia, and Lithuania

Probabilities of hip fracture were generally very similar to those in Lithuania, Poland, and Russia. At older ages, Russian probabilities rose less markedly with age and, indeed fell from the age of 85 years in women due to the competing effect of mortality (Fig. 3).

## Discussion

This study characterizes for the first time the hip fracture incidence in a region of Belarus from the age of 50 years, based on regional data from Mozyr. As expected, in both sexes incidences increased with age. Hip fractures were more common in women and from the age of 75 years, incidences were higher in women than in men. Based on age-standardized annual incidence of hip fracture women, Belarus belongs to the medium-risk countries for osteoporotic hip fracture [2].

Hip fracture rates vary markedly in different countries for reasons that are ill understood. However, the fracture rates and fracture probabilities were rather similar in Belarus compared with Russia, Lithuania, and Poland.

A disquieting finding of the present study that 29% cases of hip fracture did not come to hospital attention in Belarus. Moreover, older people and women more frequently did not undergo the necessary emergency surgery. Similar observations have been reported in Armenia [13] and Pervouralsk in Russia [14] where 44 and 27% of patients sustaining a hip fracture, respectively, did not receive specialized medical care. Similar or higher estimates are reported for Georgia (75% not hospitalized), Kazakhstan (50% not hospitalized), Kyrgyzstan (50% not hospitalized), and Moldova (uncertain proportion) [15]. The likely reason is that facilities for surgical management are limited so that hospital admission is not feasible. These “missing” cases from hospital discharge data reinforce a view that data on hip fracture based solely from hospital records are unreliable in this region of the world.

The present study also describes the FRAX model for the assessment of fracture probability in men and women from Belarus. In the absence of BMD, probabilities of a major fracture were higher than hip fracture probabilities at all ages and both increased with age in men and in women. The 10-year absolute probability of any major osteoporotic and hip fracture in the absence or presence of a prior fragility fracture increased with advancing age in both sexes, being consistently higher in women than in men. The widespread availability of FRAX has resulted in its adoption in many practice guidelines worldwide [12]. The fracture probability equivalent to a woman with a prior fracture has been used as an intervention threshold in more than 30 countries. If the same threshold were applied to Belarus, then intervention would be recommended with a probability of a major fracture that varied between 4.9 and 27% depending on age (see Table 1). The impact of such thresholds or alternative thresholds will require further study.

There are a number of limitations to this study. With regard to fracture incidence, we examined only 1.2% of the Belarus population. Therefore, the extrapolation of these regional estimations to the entire country is an assumption that we were unable to test. In addition to large variations in fracture rates around the world, fracture rates may vary within countries. In addition to ethnic-specific differences [16], up to twofold differences in hip fracture incidence have been reported using common methodology with higher rates in urban communities in Croatia [17], Switzerland [18], Norway [19], Argentina [20], and the USA [21]. The similarity in hip fracture risk in the neighboring countries of Belarus is reassuring but does not dispel the uncertainty.

With regard to the FRAX model, this was built on hip fracture rates since data on other major osteoporotic fractures were not available. As noted in the methods, the empirical data are very consistent with the methods that were used to derive fracture rates for other major fractures. Notwithstanding, there is evidence that forearm fractures in Russia and Lithuania are more common than would be predicted from hip fracture rates [14, 22], a subject that would be worthy of future investigation in Belarus.

Despite the rigor of the methodology, its prospective nature and well-defined catchment population, it is probable that not all hip fractures were captured. On the other hand, accuracy errors have little impact on the rank order with which the FRAX tool categorizes risk in a given population [13, 23], but they do change the absolute number generated and thus have implications where treatment guidelines are based on cost-effectiveness or the economic burden of disease.

In summary, a country-specific FRAX model has been developed for the Republic of Belarus based on a regional representation of the population with prospectively obtained epidemiological data. This model should enhance accuracy of determining fracture probability among the Belarus population and help to guide decisions about treatment.
